# Detection of Coronaviruses in Bats in Lebanon during 2020

**DOI:** 10.3390/pathogens12070876

**Published:** 2023-06-26

**Authors:** Ahmed Kandeil, Mounir Abi-Said, Rebecca Badra, Rabeh El-Shesheny, Ahmed A. Al-Karmalawy, Radwan Alnajjar, Zumama Khalid, Mina Nabil Kamel, Walid Abi Habib, Jad Abdallah, Vijaykrishna Dhanasekaran, Richard Webby, Ghazi Kayali

**Affiliations:** 1 Department of Infectious Diseases, St. Jude Children’s Research Hospital, Memphis, TN 38105, USA; ahmed.kandeil@stjude.org; 2 Center of Scientific Excellence for Influenza Virus, Institute of Environmental Research and Climate Change, National Research Centre, Giza 12622, Egypt; rabeh.elshesheny@human-link.org (R.E.-S.); mina@human-link.org (M.N.K.); 3 Human Link DMCC, Dubai 115738, United Arab Emirates; rebecca@human-link.org; 4 L2GE Department of Earth and Life Sciences, Faculty of Sciences II, Lebanese University, Fanar 90656, Lebanon; mabisaid9@gmail.com; 5 Pharmaceutical Chemistry Department, Faculty of Pharmacy, Ahram Canadian University, Giza 12566, Egypt; akarmalawy@acu.edu.eg; 6 Department of Chemistry, Faculty of Science, University of Benghazi, Benghazi 1308, Libya; radwan.alnajjar@uob.edu.ly; 7 Faculty of Pharmacy, Libyan International Medical University, Benghazi 16063, Libya; 8 Department of Health Sciences, University of Genova, 16126 Genova, Italy; zumama.khalid@gmail.com; 9 Multi-Omics Laboratory, School of Pharmacy, Lebanese American University, P.O. Box 36, Byblos 1401, Lebanon; waleedabihabib@gmail.com (W.A.H.); jabdallah@lau.edu.lb (J.A.); 10 School of Public Health, LKS Faculty of Medicine, The University of Hong Kong, Hong Kong 999077, China; veej@hku.hk; 11 HKU-Pasteur Pole, LKS Faculty of Medicine, The University of Hong Kong, Hong Kong 999077, China

**Keywords:** coronaviruses, SARS-CoV-2, bat, reservoir, Lebanon, zoonoses

## Abstract

Bats are considered the main reservoir of coronaviruses (CoVs), and research evidence suggests the essential role of bats in the emergence of Severe Acute Respiratory Syndrome Coronaviruses (SARS-CoV) and SARS-CoV-2. SARS-CoV-like viruses have been recently detected in bats in different countries. In 2020, we conducted surveillance for CoVs among six different bat species in Lebanon. Of 622 swab specimens taken, 77 tested positive. Alpha- and Beta- CoVs were identified in samples collected from different species. Our results show that SARS-like coronaviruses circulate in bats in this region, and we provide new data on their genetic diversity. The interaction between the spike of the detected SARS-CoV-like viruses and the human angiotensin-converting enzyme 2 (hACE2) receptor could be crucial in understanding the origin of the epidemic. The 3D protein structure analysis revealed that the receptor-binding domains of the SARS-like virus identified in Lebanon bind to the hACE2 protein more efficiently than to the spike of the SARS-CoV-2 strain. The spike of the detected SARS-CoV-like viruses does not contain the recognition site of furin at the cleavage site. Thus, our study highlights the variety of bat coronaviruses in Lebanon and suggests the zoonotic potential for other SARS-CoV-like viruses.

## 1. Introduction

After rodents, bats are the second most diverse group of mammals in the world with about 1400 different species. Bats (order Chiroptera) have recently divided mainly into Yinpterochiroptera and Yangochiroptera suborders. Bats act as important reservoir hosts for a variety of neglected and non-neglected viruses and play crucial roles in the spillover of different viruses, including emerging coronaviruses (CoVs). Some of these emerging CoVs have acquired mutations to adapt to humans directly or through intermediate hosts. Six human CoVs were characterized by the end of 2019, including CoV-OC43, CoV-229E, CoV-HKU1, CoV-NL63, Middle East Respiratory Syndrome CoV (MERS-CoV), Severe Acute Respiratory Syndrome CoV (SARS-CoV), and SARS-CoV-2. The close relationship between the six human CoVs and CoVs identified in animals supports the zoonotic origin of these viruses. The discovery of multiple SARS-CoV-related viruses in bats highlights their role in CoV transmission [1,2,3,4]. Moreover, serological evidence indicated the presence of antibodies against bat SARS-CoVs and HKU10-CoVs at a low prevalence rate among rural inhabitants in China which highlights the potential transmission of these viruses to humans [5]. Further, the COVID-19 pandemic emphasizes the significance of bat-borne viruses and the need for continued monitoring and research to understand their emergence and transmission. At early stages of the pandemic, genetic characterization of the genome of SARS-CoV-2 revealed that this newly-emerging virus was closely related to SARS-CoV and several SARS-like coronaviruses identified in bats. Later, several strains of SARS-CoV-2-related coronaviruses had been identified in pangolins and bats. Although the identification of multiple SARS-like coronaviruses strains in wildlife supports the role of wildlife in the spillover of CoVs, the evolutionary history of SARS-CoV-2 is unclear.

In Lebanon, 21 species of bats exist [6], such as Egyptian fruit bats (*Rousettus aegyptiacus*), horseshoe bats (Rhinolophus sp.), and Schreiber’s bent-winged bat (*Miniopterus schreibersii*). During the last decades, bats in their natural habitats in Lebanon have faced numerous threats, including climate change, habitat loss, hunting, and disturbance of roosting sites [7]. Understanding dynamics and diversity of CoVs in bats should be considered to prevent zoonotic transmission and add to the knowledge of virological features prior to the emergence of viruses. Our active surveillance from 2013–2015 showed the presence of HKU9-related CoV in *R. aegyptiacus* in Lebanon [8]. In this study, we aim to characterize the prevalence of CoVs in different species of bats in Lebanon during the SARS-CoV-2 pandemic.

## 2. Materials and Methods

### 2.1. Sample Collection and Processing

**Figure 1:**
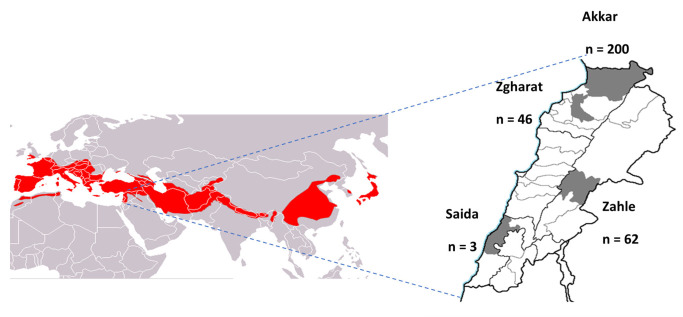
Map of Lebanon showing sample collection sites in gray and the number of samples collected in each site. The distribution of greater horseshoe bats across Eurasia is shown in red (https://commons.wikimedia.org/w/index.php?curid=12665198, accessed on 20 May 2023).

Between 27 June and 4 August 2020, we captured 311 bats from 6 different species using mist nets in mountain caves located in Akkar (n = 200), Zgharta (n = 46), Zahle (n = 62) and Saida (n = 3) (Figure 1). These included greater horseshoe bats (*Rhinolophus ferrumequinum*, n = 109), lesser horseshoe bats (*Rhinolophus hipposideros,* n = 49), Schreiber’s bent-winged bats (*Miniopterus schreibersii*, n = 51), mouse-eared bats (Myotis sp., n = 76), long-fingered bats (*Myotis capaccinii*, n = 6), and Geoffroy’s bats (*Myotis emarginatus*, n = 20). The bats were identified using external morphology and confirmed through DNA barcoding. Oral and rectal swabs were collected from each bat. Immediately after, the bats were released. The swabs were placed in a viral transport medium and transported to the laboratory on ice. The samples were then stored at −80 °C for further processing. The protocols for bat capture and sampling were approved by the Institutional Animal Care and Use Committee at St. Jude Children’s Research Hospital, USA (No. 3130).

### 2.2. Molecular Detection

Due to mismatching of the SARS-CoV-2 genome with a widely used pan-CoV primer that targets the RNA-dependent RNA polymerase (RdRp) gene [9], CoV detection was performed using a new set of degenerated primers targeting a 441bp region of the RdRp. The primers PAN-CoV-F (5′-GGGNTGGGAYTAYCCHAARTGYGA-3′) and PAN-CoV-R (5′-CNCCRTCRTCAGAHARWATCAT-3′) were designed based on 40 reference sequences from GenBank (Table S1 and Figure S1) to identify α-, β-, γ-, and δ- CoV. Primers were tested using laboratory strains of α (bat αCoV, 229E, and NL63), β (SARS-CoV-2, MERS CoV, OC43, HKU1, and HKU9), and γ (avian infectious bronchitis (IB)) CoVs.

Viral RNA was extracted from the pooled oral and rectal swabs using the QIAamp viral RNA extraction kit (Qiagen, Hilden, Germany). The extracted RNA was subjected to RT-PCR using the One-Step RT-PCR kit (Qiagen, Hilden, Germany). The PCR cycler conditions for the amplification were 50 °C for 30 min followed by 95 °C for 15 min, then 50 cycles of 94 °C for 30 s (denaturation), 50 °C for 30 s (annealing), 72 °C for 60 s (extension), then 72 °C for 10 min (final extension). PCR products of 441bp were visualized on a 1% agarose gel electrophoresis, and the targeted amplicons were purified using the QIAquick gel extraction kit (QIAgen) and sequenced at the Macrogen DNA sequencing service. The sequences were assembled using SeqMan DNA Lasergene 15 software (DNASTAR, Madison, WI, USA) and submitted to GenBank (MW880969 to MW881013).

Complete spike sequences of 3 representative samples of β-CoVs were amplified using primers designed based on BM48-31/BGR/2008 (NC 014470.1) (Table S2) and using the One-Step RT-PCR kit (Qiagen, Hilden, Germany). The PCR cycler conditions for the amplification were 50 °C for 60 min followed by 95 °C for 15 min, then 50 cycles of 94 °C for 30 s (denaturation), 50 °C for 30 s (annealing), 72 °C for 4 min (extension), then 72 °C for 10 min (final extension). Amplicons were then purified using the QIAquick gel extraction kit and sequenced at The Hartwell Center for Biotechnology, St. Jude Children’s Research Hospital. Sequences were submitted to GenBank.

### 2.3. Phylogenetic Analysis

A BLASTN analysis was conducted, and closely related sequences were downloaded from GenBank. Previously identified CoVs in Lebanon and full genome sequences of identified CoVs in bats were included. The final dataset was aligned using MAFFT v7.305b [10], manually trimmed, and phylogenetic trees were constructed using the maximum likelihood method with Kimura’s 2-parameter distance model and 1000 bootstrap using MEGA X [11].

### 2.4. Homology Modeling, Protein-Protein Docking, and Molecular Dynamic Simulation

The 3D structure of the spike protein of the Lebanon SARS-CoV-2-like virus was predicted through homology modeling, with the 3D structure of SARS-CoV-2 spike glycoprotein obtained from PDB (PDB ID: 5WRG, 4.30 Å [12] using the MOE software. The validity of the model was confirmed using the Maestro software, which resulted in an RMSD of 2.380 Å compared to the 5WRG structure. A Ramachandran plot of the novel structure was generated and is shown in Figure S2. Protein-protein docking was performed using HADDOCK [13,14]. The ACE2 receptor in complex with the spike protein (PDB ID: 6M0J) [15] was prepared by removing the spike chain and heteroatoms using the MOE software. The structures of ACE2 along with the wide and novel spike proteins were uploaded to the HADDOCK server, and the docking poses were analyzed. Only the poses that docked around the receptor binding domain (RBD) of the spike protein were considered for further analysis using PyMOL (PyMOL Molecular Graphics System, Version 1.2r3pre, Schrödinger, LLC) and UCSF Chimerax 1.5 [16]. Finally, the docked poses were subjected to a 500 ns molecular dynamic simulation to evaluate the stability and interaction between the ACE2 receptor and spike. The details of MD simulation are provided in the Supplementary Materials.

### 2.5. Statistical Analysis

Statistical analyses were performed using GraphPad Prism 9 (GraphPad, San Diego, CA, USA). The association between the prevalence of CoVs among different variables was analyzed using the Chi-square test. Statistical significance was considered at *p*-values less than 0.05.

## 3. Results

### 3.1. Prevalence of CoVs in Bats

In this study, 57 out of 311 bats from all 6 species (18.3%) tested positive for CoV, with rectal swabs showing a higher detection rate (100%) than oral swabs (35%) (*p*-value < 0.0001). The prevalence varied significantly based on swab type, collection site, species, and age (*p*-value < 0.0001) (Table 1). The highest detection rate was in *Rhinolophus ferrumequinum* (36.6%), followed by *Miniopterus schreibersii* (23.5%), *Myotis emarginatus* (10%), and *Rhinolophus hipposideros* (6.12%). The highest detection rate of CoVs was recorded at the sampling site in Saida (100%), followed by Akkar (21.5%) and Zahle (17.7%) (*p*-value < 0.0001). The detection rate was significantly higher in juvenile bats than in adult bats (*p*-value < 0.0001), but there was no difference between male and female bats (*p*-value > 0.05) (Table 1).

### 3.2. Diversity of CoVs in Lebanon Based on Partial Sequence of RdRp

**Figure 2:**
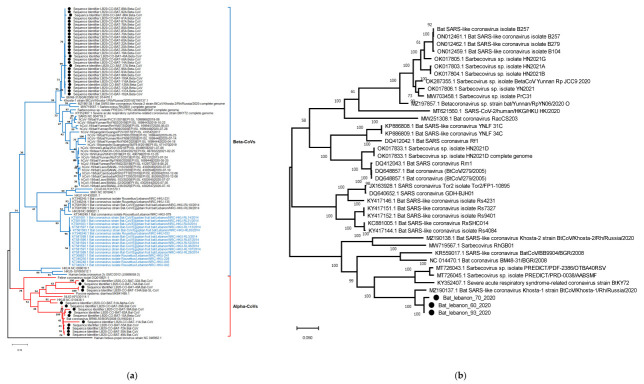
Evolutionary relationships of bat CoVs in Lebanon. Maximum likelihood phylogeny of the (**a**) RdRp gene of CoVs generated and (**b**) spike protein.

Whole genome sequencing was attempted but was not successful. Blast analysis of 42 partial sequences retrieved using Sanger sequencing showed 28 sequences were from *Rhinolophus ferrumequinum* Betacoronaviruses, and the remaining 14 were related to Alphacoronaviruses (Table S3). All Betacoronaviruses detected in this study formed a single phylogenetic cluster and were SARS-like. They were most closely related to bat coronavirus BM48-31/BGR/2008 and bat coronavirus Khosta-1, which were identified in *Rhinolophus blasii* and *Rhinolophus ferrumequinum* from Bulgaria and Russia in 2008 and 2020, respectively (Figure 2a). However, Alphacoronaviruses in Lebanon formed three divergent lineages (Figure 2a). The majority (n = 9) were most closely related to BR89-55/GBR/2008 collected from *Miniopterus schreibersii* in Germany (bootstrap, 99%), and one sequence (Bat 51A) was most closely related to the *Miniopterus* bat CoV HKU 8 (Bat-CoV HKU8) with a bootstrap of 91%, whereas the other lineage comprising four sequences was related to the Porcine epidemic diarrhea virus (PEDV).

### 3.3. Genetic and Structural Characterization of the Spike Glycoprotein of Lebanon SARS-Like CoVs and the Potential Significance of Binding to Human ACE2

**Figure 3:**
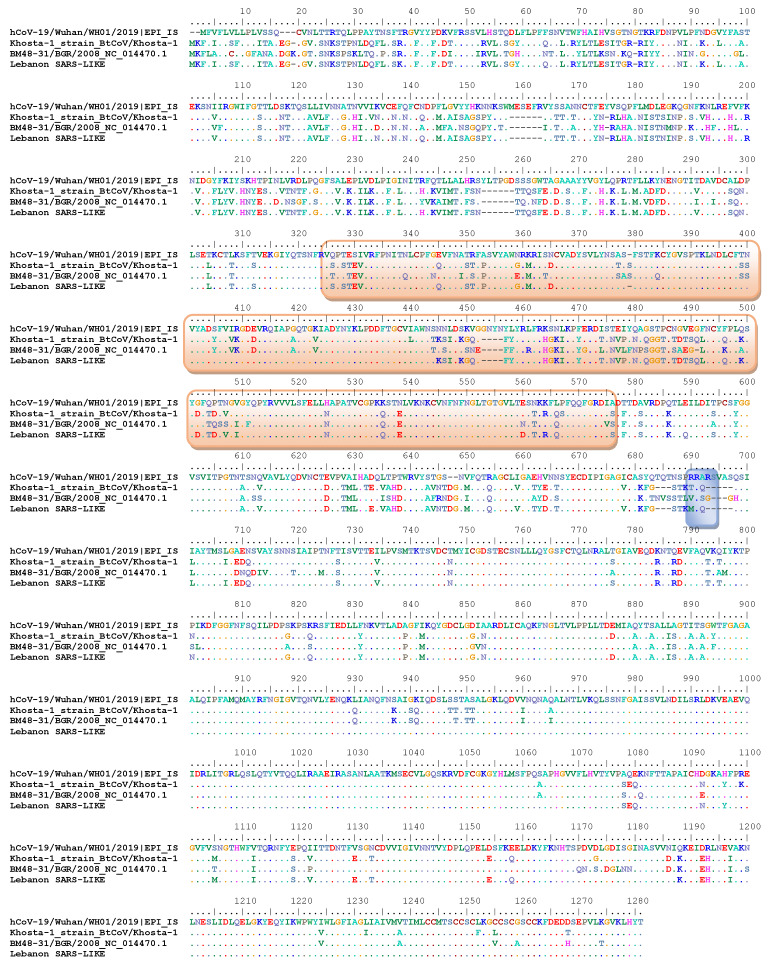
Amino acid alignment of spike protein sequences of the currently detected SARS CoV-like viruses in Lebanon and closely characterized viruses (BM48-31/BGR/2008, and bat coronavirus Khosta-1) and SARS-CoV-2.

To better understand the risk that Lebanon SARS-like CoVs pose, we sequenced the complete spike protein genes of three representative samples. They were highly similar throughout the gene (Figure 3). These spike proteins were most closely related to the bat CoV Khosta-1 strain collected during 2020 in Russia and had a 94% similarity.

A comparative analysis of the spike proteins of Lebanon Betacoroviruses and SARS-CoV, which showed five amino acid deletions at the S1/S2 junction (RQQ) in Lebanon Betacoronaviruses indicated the absence of a furin cleavage site (Figure 3). Among the five amino acids known to be critical for binding the S glycoprotein to the ACE2 receptor, (501N, 505Y, 455L, 486F, and 493Q), the 505Y and 455L amino acids were found among the Lebanon bat CoV. 

**Figure 4:**
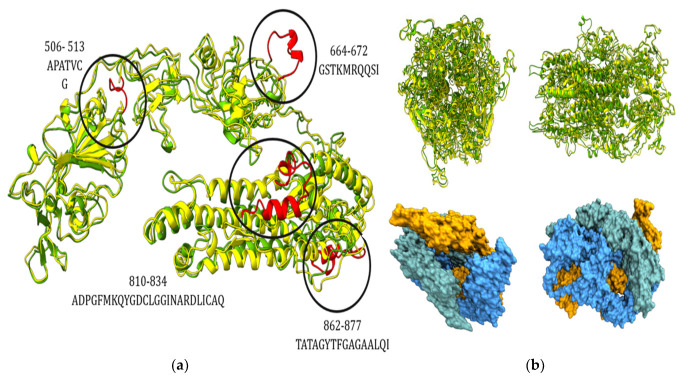
(**a**) Overlay of the 3D structure of the wild type (yellow) and novel type (green); missing residues are highlighted in red. (**b**) (Up) Overlay of the 3D structure of whole spike protein, wild (yellow) and novel (green); (down) surface representation of the novel spike protein.

Homology modeling of the chain A sequence of spike protein (Figure 4a,b and Figure S3) (Supplementary Materials) highlighted the missing regions of Lebanon bat CoV (strain name here) in residues 506-513, 664-672, 810-834, and 862-877 compared to the SARS-CoV spike protein (PDB: 5WRG).

Docking results from the HADDOCK were derived. Since minimum HADDOCK scores depicted more binding affinity of the two interacting proteins, best poses of the docking were obtained from the given results. The novel complex demonstrated significantly greater binding affinity and stability than the control complex, which had a lower HADDOCK. Z-score indicated how many standard deviations from the average the resultant complex was located in terms of score (the more negative, the better). Docking scores of both complexes along with other parameters are demonstrated in Table 2.

Next, the interactions between the spike protein (wild and novel) and the ACE2 were analyzed. The H-bond interactions, along with their residues and distances, are reported in Table 3. In contrast to the wild-type spike, which formed 14 H-bonds with the ACE2 receptor (Table 3), 8 H-bonds were formed between ACE2 and chain C, while 6 were formed with chain A. On the other hand, the novel protein spike was able to form 22 H-bonds with the ACE2 receptor: 13 H-bonds were formed via interaction with chain C, while the other nine H-bonds were formed with chain B. The interactions of both wild and novel proteins are presented in Figures S3–S8.

**Figure 5:**
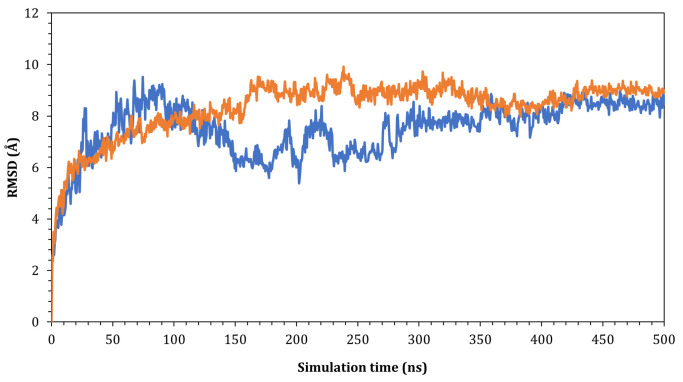
The RMSD for Cα atoms (Å) with respect to the initial structure as a function of simulation time (ns) for the complexes. Blue SARS-CoV2 and orange are the newly detected viruses in Lebanon.

To validate the results obtained from docking, dynamic molecular simulation was implemented to study the stability of the wild-ACE2 and novel spike-ACE2 complexes. Our focus was only on the interactions between the ACE2 and receptor binding domain. Hence, only the RBD was considered for the MD calculations, and both complexes were subject to a 500 ns simulation. The root mean square deviation of the C𝛼 of the proteins was plotted as a function of simulation time to measure the impact of the simulation on the stability of the SARS-CoV-2 spike-ACE2 complexes. As seen in Figure 5, the wild SARS-CoV-2 spike-ACE2 complex fluctuated at 9.50 Å with respect to its original C𝛼 atoms at the start of the simulation time. The complex further oscillated at around 3.00 Å until around 300 ns of the simulation time, and, at around 350 ns, the complex reached a plateau and fluctuated at around 1.00 Å until the end of the simulation. On the other hand, the novel SARS-CoV-2 spike-ACE2 complex showed a similar pattern but reached stability earlier at around 180 ns of the simulation time. The complex fluctuated at around 9.00 Å at the beginning of the simulation and reached stability at 180 ns, and the complex fluctuated at around 1.00 Å toward the end of the simulation.

The H-bond interactions were monitored during the simulation, and snapshots of the spike-ACE2 were taken at each 50 ns (Table 4). As seen in Table 4, the complex holds around 15-22 H-bonds during the simulation. The residues involved in the formation of these interactions are reported in detail in Table S4.

## 4. Discussion

Horseshoe bats (*Rhinolophidae*) are considered potential reservoir hosts for several viruses that can cause disease in humans and animals. They are widely distributed in Asia, Europe, and North Africa. In East and Southeast Asia, SARS-CoV-like viruses have been found in multiple rhinolophids [1,3,17]. Recently, SARS-like CoVs have been identified in Rhinolophus sp. in Russia during 2020 [18]. However, information about the prevalence and genetic characteristics of CoVs in Middle Eastern bats is limited. To address this, we conducted a cross-sectional surveillance for CoVs among six bat species in Lebanon during 2020. Not surprisingly, we identified several Alpha- and Betacoronaviruses in samples collected from different species of Microchiroptera in Lebanon. However, the zoonotic origin of the newly-emerging coronaviruses (SARS-CoV, SARS-CoV-2, and MERS-CoV) remains unclear. Moreover, there are genetic differences among detected viruses in humans and animals, thus supporting the presence of intermediate hosts or missing genetic information. However, the limitations of this study include the lack of virus isolation in culture and whole genome sequences, as well as in vitro data on binding and serology.

The closely genetically-related β-CoVs detected in bats with SARS-CoV-2 (NC_045512) were RaTG13 (in *Rhinolophus affinis*), and RpYN06 (in *Rhinolophus pusillus*) viruses in China with sequence identities 96.10 and 94.48%, respectively, across full genomes [2]. However, the spike gene has a much lower sequence identity, suggestive of a genomic recombination event, thus making RpYN06 the second closest relative of SARS-CoV-2 identified to date after the bat CoV RaTG13, hence highlighting the potentially complex evolutionary history of SARS-CoV-2 [2,4]. Other SARS-CoV-2-related CoVs such as RmYN02, STT182 and STT200 were detected in *R. malayanus* and *R. shameli* bats in Asian countries [1,17].

However, for emerging zoonotic spillovers, humans exposed to the viruses should be infected through direct contact with the infected reservoir or intermediate host of coronaviruses. Other factors such as environmental factors play a role in emerging zoonotic viruses. In Lebanon, there is almost no direct contact between humans and bats, and the intensity of the reservoir bat–human interface is low, which decreases the potential for spillover risk. Globally, extensive studies of the genetic diversity of coronavirus in bats could bridge our knowledge gaps associated with the emergence of SARS-CoV-2.

In line with previous studies [2], analysis of the 3D protein structure revealed that the receptor-binding domains of the SARS-like virus identified in Lebanon bind more efficiently to the hACE2 protein than to the spike of the SARS-CoV-2 strain. Risk assessment of viruses that are potentially infectious to humans should be prioritized by national, regional, and international agencies. In summary, our results suggest a diverse range of CoVs in bats, with Betacoronaviruses and Alphacoronaviruses co-existing in the same species. The high prevalence of CoVs in *Rhinolophus ferrumequinum,* in particular, underscores the importance of continued surveillance and investigation of this species in understanding CoV emergence. Furthermore, our results underline the importance of continued monitoring of CoV in bats, especially in SARS-like Betacoronaviruses that have repeatedly emerged in humans.

Surveillance for coronaviruses in bats is critical as an alarm system for emerging and remerging viruses through early detection, monitoring transmission dynamics, identifying high-risk species or regions, tracking genetic diversity, and informing public health policies.

## Figures and Tables

**Table 1:** Detection of CoVs in bats in Lebanon.

Variable	Number of Bats (Percentage of Total Bats)	Number Positive (% Samples in Each Category)	*p*-Value *
**Sample type**			
Oral	311 (50%)	20 (6.4%)	<0.0001
Rectal	311 (50%)	57 (18.3%)	
**Type of bat**			
*Miniopterus schreibersii*	51 (16.4%)	12 (23.5%)	<0.0001
*Myotis emarginatus*	20 (6.4%)	2 (10%)	
*Myotis capaccinii*	6 (1.9%)	0 (0%)	
*Myotis* sp.	76 (24.4%)	0 (0%)	
*Rhinolophus ferrumequinum*	109 (35.0%)	40 (36.6%)	
*Rhinolophus hipposideros*	49 (15.8%)	3 (6.1%)	
**Governorates**			
Akkar	200 (64.3%)	43 (21.5%)	<0.0001
Saida	3 (1.0%)	3 (100%)	
Zahle	62 (19.9%)	11 (17.7%)	
Zgharat	46 (14.8%)	0 (0%)	
**Gender**			
Male	140 (45%)	28 (20%)	NS
Female	171 (55%)	29 (16.9%)	
**Age**			
Adult	288 (92.6%)	43 (14.9%)	<0.0001
Juvenile	23 (7.3%)	14 (60.8%)	

* *p*-value was obtained using Chi-square test; NS, not significant.

**Table 2:** Docking results of control complex (ACE2 and 5WRG) and novel complex. Docking scores are represented by HADDOCK scores (the more negative docking scores, the better the binding affinity of two proteins).

Complex	HADDOCK Score	Cluster Size	RMSD from the Overall Lowest-Energy Structure	Z-Score	Van der Waals Energy
CNT	−104.2 ± 6.7	31	1.3 ± 1.1	−1.7	−83.0 ± 1.8
Novel	−206.6 ± 15.9	15	0.5 ± 0.4	−2.4	−81.0 ± 12.9

**Table 3:** H-bond interactions between the spike of the wild and the novel spike.

Wild		ACE2	Distance (Å)	Novel		ACE2	Distance (Å)
Chain C	Thr320	Asp597	3.01	Chain C	Asn324	Glu564	3.00
	Asn321	Lys596	3.12		Cys326	Gln388	3.17
	Leu322	Lys596	3.08		Gln330	Gln388	3.50
	Asn330	Glu238	2.93		Gln330	Pro389	3.35
	Asp351	Asn601	2.91		Gln330	Arg559	2.53
	Thr359	Asp615	3.13		Ser349	Gln89	2.71
Chain B	Tyr440	Pro253	2.82		Cys351	Glu22	3.35
	Asn479	Asp157	3.08		Cys351	Asn90	3.81
	Asn479	Asn159	3.29		Asp354	Lys26	3.19
	Asp480	Tyr255	2.79		Asp354	Asn90	2.84
	Thr487	Tyr613	3.23		Tyr359	Asp30	2.91
	Gly488	Ala614	3.44		Asp486	Gln325	2.92
Chain C	Lys514	Gln598	2.70		Cys512	Gln89	2.72
	Lys514	Asn601	2.57	Chain B	Ile432	Lys68	2.45
					Lys434	Glu75	2.75
					Ala462	Met82	3.29
					Leu473	Gln81	3.19
					Leu473	Met82	3.77
					Cys475	Leu79	3.55
					Cys475	Gln81	3.55
					Tyr476	Glu75	2.77
					Tyr482	Glu35	3.07

**Table 4:** H-bonds interactions between the SARS-CoV-2 spike and ACE 2 during simulations.

Simulation Time (ns)	0	50	100	150	200	250	300	350	400	450	500
H-bonds	20	15	19	22	20	21	22	20	19	22	22

## Data Availability

All data are present in the manuscript and its Supplementary Materials.

## Supplementary Materials

The following supporting information can be downloaded at: https://www.mdpi.com/article/10.3390/pathogens12070876/s1, Figure S1. Graphic illustration of the selected conserved regions that was created via a web-based WebLogo application (http://weblogo.threeplusone.com/create.cgi, accessed on 20 May 2023); Figure S2. The Ramachandran plot of the novel SAR-CoV-2 spike; Figure S3. Validation of the homology modelling results of the novel SAR-CoV-2 spike; Figure S4. Sequence alignment of the wild and the novel spike proteins; Figure S5. Wild-ACE (green) interaction, Figure S6. Novel-ACE2 (green) interaction; Figure S7. Wild-ACE2 interactions; Figure S8. Novel-ACE2 interactions; Table S1. Representative α-, β-, γ-, and δ- CoV sequences retrieved from National Center for Biotechnology Information (NCBI) that was used for degenerated primers design; Table S2. Primers used for sequencing of the spike; Table S3. Nucleotide sequence identities between partial RdRp sequences detected in bats in Lebanon and nearest published sequences in GenBank based on BLASTN analysis; Table S4. Interactions between the novel spike and ACE2 receptor at different simulation times.

## References

[1] Delaune D., Hul V., Karlsson E.A., Hassanin A., Ou T.P., Baidaliuk A., Gambaro F., Prot M., Tu V.T., Chea S. (2021). A novel SARS-CoV-2 related coronavirus in bats from Cambodia. Nat. Commun..

[2] Zhou H., Ji J., Chen X., Bi Y., Li J., Wang Q., Hu T., Song H., Zhao R., Chen Y. (2021). Identification of novel bat coronaviruses sheds light on the evolutionary origins of SARS-CoV-2 and related viruses. Cell.

[3] Wacharapluesadee S., Tan C.W., Maneeorn P., Duengkae P., Zhu F., Joyjinda Y., Kaewpom T., Chia W.N., Ampoot W., Lim B.L. (2021). Author Correction: Evidence for SARS-CoV-2 related coronaviruses circulating in bats and pangolins in Southeast Asia. Nat. Commun..

[4] Zhou P., Yang X.L., Wang X.G., Hu B., Zhang L., Zhang W., Si H.R., Zhu Y., Li B., Huang C.L. (2020). A pneumonia outbreak associated with a new coronavirus of probable bat origin. Nature.

[5] Li H., Mendelsohn E., Zong C., Zhang W., Hagan E., Wang N., Li S., Yan H., Huang H., Zhu G. (2019). Human-animal interactions and bat coronavirus spillover potential among rural residents in Southern China. Biosaf. Health.

[6] Benda P., Abi-Said M., Bou Jaoude I., Ka R., Lučan R., Sadek R., Ševčík M., Uhrin M., Horacek I. (2016). Bats (Mammalia: Chiroptera) of the Eastern Mediterranean and Middle East. Part 13. Review of distribution and ectoparasites of bats in Lebanon. Acta Soc. Zool. Bohem..

[7] Abi-Said M. (2014). Monitoring, threats and conservation of hibernating bats roosts in Lebanon. Jordan J. Nat. Hist..

[8] Shehata M.M., Chu D.K., Gomaa M.R., AbiSaid M., El Shesheny R., Kandeil A., Bagato O., Chan S.M., Barbour E.K., Shaib H.S. (2016). Surveillance for Coronaviruses in Bats, Lebanon and Egypt, 2013–2015. Emerg. Infect. Dis..

[9] Watanabe S., Masangkay J.S., Nagata N., Morikawa S., Mizutani T., Fukushi S., Alviola P., Omatsu T., Ueda N., Iha K. (2010). Bat coronaviruses and experimental infection of bats, the Philippines. Emerg. Infect. Dis..

[10] Katoh K., Standley D.M. (2013). MAFFT multiple sequence alignment software version 7: Improvements in performance and usability. Mol. Biol. Evol..

[11] Kumar S., Stecher G., Li M., Knyaz C., Tamura K. (2018). MEGA X: Molecular Evolutionary Genetics Analysis across Computing Platforms. Mol. Biol. Evol..

[12] Gui M., Song W., Zhou H., Xu J., Chen S., Xiang Y., Wang X. (2017). Cryo-electron microscopy structures of the SARS-CoV spike glycoprotein reveal a prerequisite conformational state for receptor binding. Cell Res..

[13] van Zundert G.C.P., Rodrigues J.P.G.L.M., Trellet M., Schmitz C., Kastritis P.L., Karaca E., Melquiond A.S.J., van Dijk M., de Vries S.J., Bonvin A.M.J.J. (2016). The HADDOCK2.2 Web Server: User-Friendly Integrative Modeling of Biomolecular Complexes. J. Mol. Biol..

[14] Honorato R.V., Koukos P.I., Jiménez-García B., Tsaregorodtsev A., Verlato M., Giachetti A., Rosato A., Bonvin A.M.J.J. (2021). Structural Biology in the Clouds: The WeNMR-EOSC Ecosystem. Front. Mol. Biosci..

[15] Lan J., Ge J., Yu J., Shan S., Zhou H., Fan S., Zhang Q., Shi X., Wang Q., Zhang L. (2020). Structure of the SARS-CoV-2 spike receptor-binding domain bound to the ACE2 receptor. Nature.

[16] Pettersen E.F., Goddard T.D., Huang C.C., Couch G.S., Greenblatt D.M., Meng E.C., Ferrin T.E. (2004). UCSF Chimera—A visualization system for exploratory research and analysis. J. Comput. Chem..

[17] Zhou H., Chen X., Hu T., Li J., Song H., Liu Y., Wang P., Liu D., Yang J., Holmes E.C. (2020). A Novel Bat Coronavirus Closely Related to SARS-CoV-2 Contains Natural Insertions at the S1/S2 Cleavage Site of the Spike Protein. Curr. Biol..

[18] Alkhovsky S., Lenshin S., Romashin A., Vishnevskaya T., Vyshemirsky O., Bulycheva Y., Lvov D., Gitelman A. (2022). SARS-like Coronaviruses in Horseshoe Bats (*Rhinolophus* spp.) in Russia, 2020. Viruses.

