# Supplementary material for: Detection of Coronaviruses in Bats in Lebanon during 2020
Source: Pathogens. 2023 Jun 26;12(7):876. doi: 10.3390/pathogens12070876 (PMC10383746; doi:10.3390/pathogens12070876)
Supplement: Supplementary file 1 [file pathogens-12-00876-s001.zip › pathogens-2441765-supplementary.pdf]

Supplementary File

Table S1. Representative α-, β-, γ-, and δ- CoV sequences retrieved from National Center for Biotechnology Information (NCBI) that used for degenerated primers design.

Strain	Accession no.
Rousettus bat coronavirus HKU9, complete genome	NC_009021.1
Bat SARS-like coronavirus RsSHC014, complete genome	KC881005.1
Bat SARS-like coronavirus Rs3367, complete genome	KC881006.1
SARS-like coronavirus BatCoV/BB9904/BGR/2008 spike glycoprotein gene, complete cds	KR559017.1
Bat SARS Cov Rs806/2006 spike protein gene, complete cds	FJ588692.1
Middle East respiratory syndrome-related coronavirus isolate MERS-CoV camel/Kenya/C1272/2018, complete genome	MH734115.1
Murine hepatitis virus strain ML-11	AF207902.1
Bovine coronavirus isolate BCoV_2014_13, complete genome	KX982264.1
Human coronavirus OC43 strain HCoV-OC43/UK/London/2011, complete genome	KU131570.1
Human coronavirus HKU1 strain HKU1/human/USA/HKU1-18/2010, complete genome	KF430201.1
Coronavirus IBV gene for spike protein precursor (infectious bronchitis virus)	X02342.1
Porcine deltacoronavirus strain PDCV_ZJ17HZ0102 spike protein mRNA, complete cds	MF461412.1
Human coronavirus 229E isolate HCoV-229E/BN1/GER/2015, complete genome	KU291448.1
Human coronavirus NL63 strain NL63/human/USA/891-4/1989, complete genome	KF530114.1
Porcine epidemic diarrhea virus isolate PEDV YZ, complete genome	MK841495.1
Transmissible gastroenteritis virus strain TGEV AHHF, complete genome	KX499468.1
Feline coronavirus strain FIPV 79-1146, complete genome	DQ010921.1
Sable antelope coronavirus US/OH1/2003	EF424621.1
Bottlenose dolphin coronavirus HKU22 isolate CF090331, complete genome	KF793826.1
Bulbul coronavirus HKU11-934	FJ376619.2
Beluga Whale coronavirus SW1	NC_010646.1
Common-moorhen coronavirus HKU21	NC_016996.1
Dromedary camel coronavirus HKU23	KF906251.1
Betacoronavirus Erinaceus/VMC/DEU/2012	NC_039207.1
Feline infectious peritonitis virus	AY994055.1
Hipposideros bat coronavirus HKU10 isolate LSH5A	JQ989269.1
Avian infectious bronchitis virus partridge/GD/S14/2003	AY646283.1
Avian infectious bronchitis virus isolate Peafowl/GD/KQ6/2003	AY641576.1
Mouse hepatitis virus strain MHV-A59 C12	NC_001846.1
Bat coronavirus HKU8	NC_010438.1
Magpie-robin coronavirus HKU18	NC_016993.1
Munia coronavirus HKU13-3514	FJ376622.1
Night-heron coronavirus HKU19	NC_016994.1
Porcine epidemic diarrhea virus	NC_003436.1
Porcine hemagglutinating encephalomyelitis virus	NC_007732.1
Wigeon coronavirus HKU20	NC_016995.1
Scotophilus bat coronavirus 512	NC_009657.1
Sparrow coronavirus HKU17	NC_016992.1
Turkey coronavirus	NC_010800.1
Thrush coronavirus HKU12-600	FJ376621.1
covid-19/NRC-3/2020	EPI_ISL_430820

Table S2. Primers used for sequencing of the spike.

Primer name	Sequences
spike 1F	CCTTTGACATGAGTAAATTTCC
Spike 1R	GTTTTGTGAACAATCTACGGCAT
spike 2F	TATACTACTGCTACCAACTGCAC
Spike 2R	TCACTAATGCGCATTCTCCCC

spike 3F	AGAGTTTCTCCTAGTACTGAAG
Spike 3R	CAGGTGTGATAACACTGACACC
spike 4F	AATGGACTCACAGGCACAGGTGT
Spike 4R	CAGCAAAGGGGGTAGAACTGTTAAGCC
spike 5F	TGTCGAACAAGACAGAAATACTAGGG
Spike 5R	GTCGTCTTCATCAAATTTGCAGCAGG
spike 6F	TTAGAGCTGCAGAAATCAGAGC
Spike 6R	CTCCTTTGAGCACTGGCTCAGA

Table S3. Nucleotide sequence identities between partial RdRp sequences detected in bats in Lebanon and nearest published sequences in GenBank based on BLASTN analysis.

Sequence identifier	GenBank Accession Number	Relative strain	GenBank Accession Number	Identity to matched sequence (%)
LB20-CO-BAT-37A.Beta-CoV	MW880969	Betacoronavirus BtCoV/Rhi_bla/BB98-22/BGR/2008	KC633201.1	97
LB20-CO-BAT-86A.Beta-CoV	MW880970	Betacoronavirus BtCoV/Rhi_bla/BB98-22/BGR/2008	KC633201.1	98
LB20-CO-BAT-46A.Beta-CoV	MW880971	Betacoronavirus BtCoV/Rhi_bla/BB98-22/BGR/2008	KC633201.1	98
LB20-CO-BAT-92A.Beta-CoV	MW880972	Betacoronavirus BtCoV/Rhi_bla/BB98-22/BGR/2008	KC633201.1	98
LB20-CO-BAT-137A.Beta-CoV	MW880973	Betacoronavirus BtCoV/Rhi_bla/BB98-22/BGR/2008	KC633201.1	98
LB20-CO-BAT-11A.Bat-CoV	MW880974	Bat coronavirus BR98-55/BGR/2008	GU190240.1	96
LB20-CO-BAT-82A.Bat-CoV	MW880975	Bat coronavirus BR98-55/BGR/2008	GU190240.1	97
LB20-CO-BAT-100A.Beta-CoV	MW880976	Bat coronavirus BM48-31/BGR/2008	KC633201.1	98
LB20-CO-BAT-51A.Alpha-CoV	MW880977	Alphacoronavirus sp. isolate 11F	MN701038.1	98
LB20-CO-BAT-78A.Beta-CoV	MW880978	Betacoronavirus BtCoV/Rhi_bla/BB98-22/BGR/2008	KC633201.1	98
LB20-CO-BAT-116A.Beta-CoV	MW880979	Betacoronavirus BtCoV/Rhi_bla/BB98-22/BGR/2008	KC633201.1	98
LB20-CO-BAT-32A.Bat-CoV	MW880980	Bat coronavirus BM48-39/BGR/2008	GU190234.1	99
LB20-CO-BAT-74A.Bat-CoV	MW880981	Bat coronavirus BM48-39/BGR/2008	GU190234.1	99
LB20-CO-BAT-58A.Beta-CoV	MW880982	Betacoronavirus BtCoV/Rhi_bla/BB98-22/BGR/2008	KC633201.1	98
LB20-CO-BAT-80A.Beta-CoV	MW880983	Betacoronavirus BtCoV/Rhi_bla/BB98-22/BGR/2008	KC633201.1	98
LB20-CO-BAT-14A.Beta-CoV	MW880984	Betacoronavirus BtCoV/Rhi_bla/BB98-22/BGR/2008	KC633201.1	97
LB20-CO-BAT-29A.Bat-CoV	MW880986	Bat coronavirus BR98-55/BGR/2008	GU190240.1	98
LB20-CO-BAT-162A.Beta-CoV	MW880987	Betacoronavirus BtCoV/Rhi_bla/BB98-22/BGR/2008	KC633201.1	98
LB20-CO-BAT-93A.Beta-CoV	MW880988	Betacoronavirus BtCoV/Rhi_bla/BB98-22/BGR/2008	KC633201.1	98
LB20-CO-BAT-70A.Beta-CoV	MW880989	Betacoronavirus BtCoV/Rhi_bla/BB98-22/BGR/2008	KC633201.1	98
LB20-CO-BAT-132A.Beta-CoV	MW880990	Betacoronavirus BtCoV/Rhi_bla/BB98-22/BGR/2008	KC633201.1	98
LB20-CO-BAT-56A.Beta-CoV	MW880991	Betacoronavirus BtCoV/Rhi_bla/BB98-22/BGR/2008	KC633201.1	98
LB20-CO-BAT-15A.Beta-CoV	MW880992	Bat coronavirus BR98-55/BGR/2008	GU190240.1	97
LB20-CO-BAT-22A.Beta-CoV	MW880993	Betacoronavirus BtCoV/Rhi_bla/BB98-22/BGR/2008	KC633201.1	98
LB20-CO-BAT-134A.Bat-SL-CoV	MW880994	BtMs-AlphaCoV/GS2013	KJ473810.1	97
LB20-CO-BAT-96A.Beta-CoV	MW880995	Betacoronavirus BtCoV/Rhi_bla/BB98-22/BGR/2008	KC633201.1	98
LB20-CO-BAT-85A.Beta-CoV	MW880996	Betacoronavirus BtCoV/Rhi_bla/BB98-22/BGR/2008	KC633201.1	98
LB20-CO-BAT-39A.Bat-CoV	MW880997	Bat coronavirus BR98-55/BGR/2008	GU190240.1	95
LB20-CO-BAT-91A.Beta-CoV	MW880998	Betacoronavirus BtCoV/Rhi_bla/BB98-22/BGR/2008	KC633201.1	98
LB20-CO-BAT-26A.Beta-CoV	MW880999	Betacoronavirus BtCoV/Rhi_bla/BB98-22/BGR/2008	KC633201.1	98
LB20-CO-BAT-30A.Bat-CoV	MW881000	Bat coronavirus BR98-55/BGR/2008	GU190240.1	97
LB20-CO-BAT-49A.Bat-CoV	MW881002	Bat coronavirus BR98-55/BGR/2008	GU190240.1	99
LB20-CO-BAT-55A.Bat-CoV	MW881003	Bat coronavirus BR98-55/BGR/2008	GU190240.1	99
LB20-CO-BAT-60A.Beta-CoV	MW881004	Betacoronavirus BtCoV/Rhi_bla/BB98-22/BGR/2008	KC633201.1	98
LB20-CO-BAT-72A.Bat-CoV	MW881005	Bat coronavirus BR98-55/BGR/2008	GU190240.1	99
LB20-CO-BAT-19A.Beta-CoV	MW881006	Betacoronavirus BtCoV/Rhi_bla/BB98-22/BGR/2008	KC633201.1	98
LB20-CO-BAT-89A.Beta-CoV	MW881007	Betacoronavirus BtCoV/Rhi_bla/BB98-22/BGR/2008	KC633201.1	98
LB20-CO-BAT-87A.Beta-CoV	MW881008	Betacoronavirus BtCoV/Rhi_bla/BB98-22/BGR/2008	KC633201.1	98
LB20-CO-BAT-98A.Beta-CoV	MW881009	Betacoronavirus BtCoV/Rhi_bla/BB98-22/BGR/2008	KC633201.1	98
LB20-CO-BAT-20A.Beta-CoV	MW881011	Betacoronavirus BtCoV/Rhi_bla/BB98-22/BGR/2008	KC633201.1	98
LB20-CO-BAT-40A.Bat-CoV	MW881012	BtMs-AlphaCoV/GS2013	KJ473810.1	97
LB20-CO-BAT-126A.Beta-CoV	MW881013	Betacoronavirus BtCoV/Rhi_bla/BB98-22/BGR/2008	KC633201.1	98

Table S4. Interactions between the novel spike and ACE2 receptor at different simulation times.

0ns		50ns		100ns		150ns		200ns	
**SetA**	**SetB**	**SetA**	**SetB**	**SetA**	**SetB**	**SetA**	**SetB**	**SetA**	**SetB**
Asn324	Glu564	Arg318	Asp213	Arg318	Asp213	Arg318	Asp213	Arg318	Asp213
Gln330	Gln388	Asn324	Ser563	Gln330	Met383	Asn324	Ser563	Asn324	Thr92
Gln330	Arg559	Asn324	Glu564	Gln330	Ala387	Asn324	Glu564	Cys326	Gln388
Cys351	Gln89	Gln330	Met383	Asp350	Asn90	Cys326	Ala387	Gln330	Met383
Cys351	Asn90	Gln330	Ala387	Asp354	Lys26	Cys326	Gln388	Gln330	Ala387
Asp354	Lys26	Asp354	Lys26	Asp354	Asn90	Gln330	Met383	Asp350	Asn90
Asp354	Asn90	Asp354	Asn90	Tyr355	Ala387	Gln330	Ala387	Asp354	Lys26
Asp354	Thr92	Tyr355	Ala387	Tyr359	Asp30	Asn333	Gly319	Asp354	Asn90
Tyr359	Asp30	Tyr359	Asp30	Lys377	Glu23	Asp350	Asn90	Tyr359	Asp30
Asn360	Asp30	Ser431	Asn322	Lys515	Glu22	Asp354	Lys26	Lys515	Glu22
Asn360	Ala387	Asp433	Asn322	Gln661	Gln24	Asp354	Asn90	Lys515	Gln89
Ser431	Asn322	Lys515	Gln89	Gly663	Ser19	Tyr359	Asp30	Ser517	Gln89
Asp486	Gln325	Asp667	Ser19	Asp667	Ser19	Lys377	Glu23	Gln550	Asp213
Ala658	Ser19	Tyr732	Thr78	Phe699	Met82	Asn428	Met383	Gln656	Ser19
Ile688	Lys68	Lys736	Glu75	Gln719	Arg192	Lys515	Glu22	Gln661	Tyr83
Lys690	Glu75			Ser727	Val107	Gln656	Ser19	Thr662	Ser19
Gln728	Gln101			Tyr732	Glu75	Gln661	Lys31	Thr662	Gln24
Leu729	Gln81			Tyr732	Thr78	Thr662	Ser19	Cys731	Thr78
Cys731	Thr78			Lys736	Glu75	His701	Gln81	Cys731	Met82
Cys731	Gln81					Gln728	His195	Tyr732	Thr78
						Tyr732	Thr78		
						Gln733	Met82		

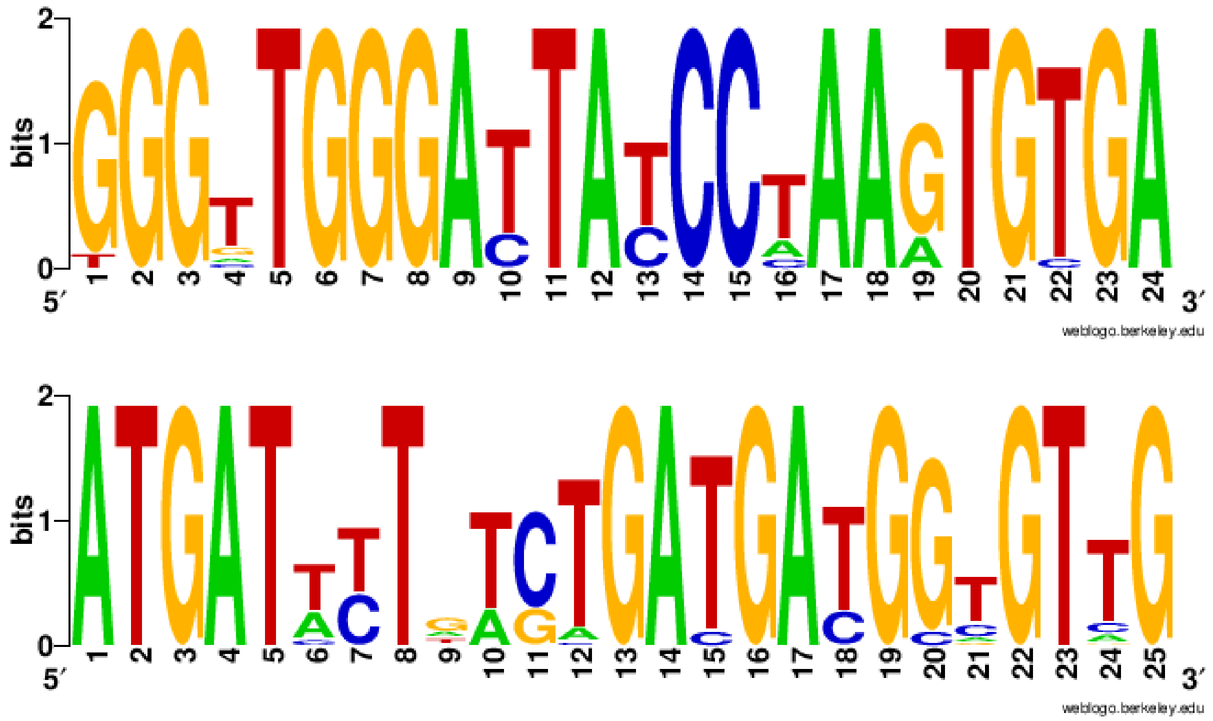

Figure S1. Graphic illustration of the selected conserved regions that was created via a Web-based WebLogo application (http://weblogo.threeplusone.com/create.cgi).

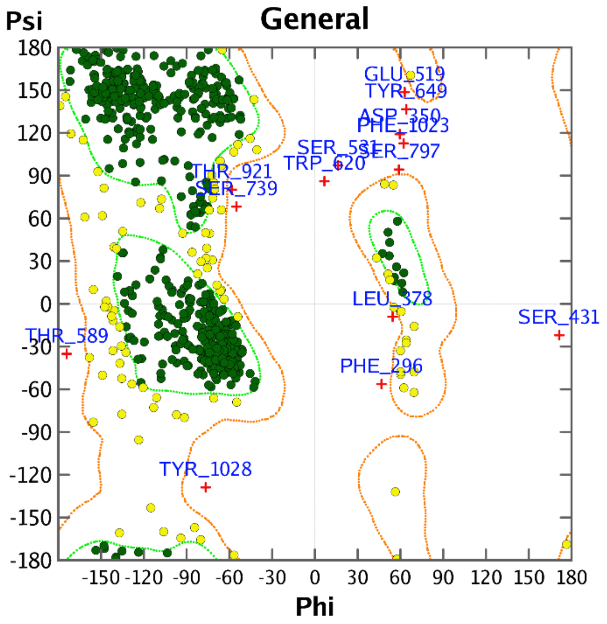

Figure S2. the Ramachandran plot of the novel SAR-CoV-2 spike.

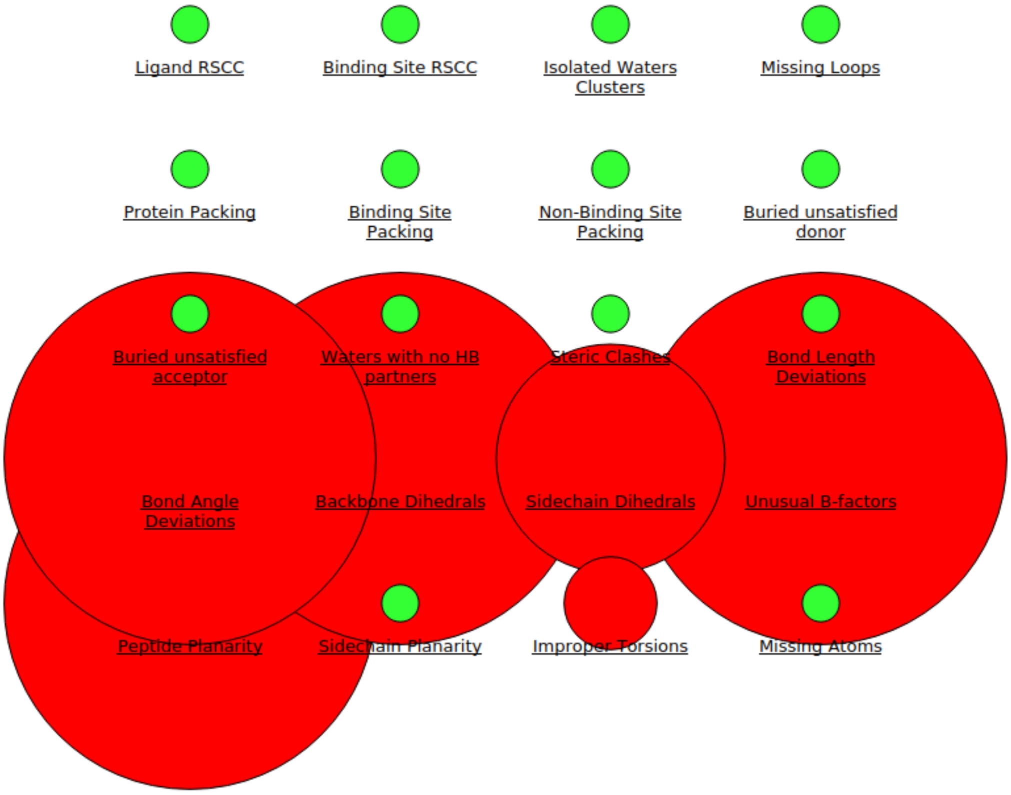

Figure S3. Validation of the homology modelling results of the novel SAR-CoV-2 spike.

```
Query  4    FIFLSFLPLITAQE--GCGVLSNKSTPNLDQFLSSKRGFYYFDDTFRSSVRVLTSGYFLP  61
FIFL FL L + +  C   +  PN  Q  SS RG YY D+ FRS    LT   FLP
Sbjct  2    FIFLLFLTLTSGSDLDRCTTFDDVQAPNYTQHTSSMRGVYYPDEIFRSDTLYLTQDLFLP  61

Query  62   FQSNLTRYLTLESITGRRIYFDNPNIPFKDGLYFAATEKSNVIRGWIFGSTLDNTTQSAV  121
            F SN+T + T+        F NP IPFKDG+YFAATEKSNV+RGW+FGST++N +QS +
Sbjct  62   FYSNVTGFHTINHT------FGNPVIPFKDGIYFAATEKSNVVRGWVFGSTMNNKSQSVI  115

Query  122  LFNNGTHIVINVCNFNFCQDPMLAISA--GSPYKSWVYTTATNCTYNRLH-AFNISTNIN  178
            + NN T++VI  CNF  C +P  A+S   G+   + ++  A NCT+  +  AF++ +
Sbjct  116  IINNSTNVVIRACNFELCDNPFFAVSKPMGTQTHTMIFDNAFNCTFEYISDAFSLDVSEK  175

Query  179  PGSFVHLREHVFRNVDGFLYVYHNYESINVTNTFPGGFSVLKPILKLPFGLNITHFKVIM  238
             G+F HLRE VF+N DGFLYVY  Y+ I+V    P GF+ LKPI KLP G+NIT+F+ I+
Sbjct  176  SGNFKHLREFVFKNKDGFLYVYKGYQPIDVVRDLPSGFNTLKPIFKLPLGINITNFRAIL  235

Query  239  TLFSNTTQSFEADASAYFVGHLKPLTMLADFDENGTIVDAVDCSQNPLSELKCTTKSFSV  298
            T FS    +  A+AYFVG+LKP T +  +DENGTI DAVDCSQNPL+ELKC+ KSF +
Sbjct  236  TAFSPAQDIWGTSAAAYFVGYLKPTTFMLKYDENGTITDAVDCSQNPLAELKCSVKSFEI  295

Query  299  EKGIYQTSNFRVSPSTEVVRFPNITNLCPFGQVFNASTFPSVYAWGRMRISDCVADYSVL  358
            +KGIYQTSNFRV PS +VVRFPNITNLCPFG+VFNA+ FPSVYAW R +IS+CVADYSVL
Sbjct  296  DKGIYQTSNFRVVPSGDVVRFPNITNLCPFGEVFNATKFPSVYAWERKKISNCVADYSVL  355

Query  359  YNSTSSFSTFKCYGVSPTKLNDLCFSSVYADYFVVKGDDVRQIAPAQTGVIADYNYKLPD  418
            YNST  FSTFKCYGVS TKLNDLCFS+VYAD FVVKGDDVRQIAP QTGVIADYNYKLPD
Sbjct  356  YNSTF-FSTFKCYGVSATKLNDLCFSNVYADSFVVKGDDVRQIAPGQTGVIADYNYKLPD  414

Query  419  DFTGCVLAWNTKSIDKGQ----GFYYRLFRHGKIKPYERDTSNVPYNAQGGTCTDTSQLN  474
            DF GCVLAWNT++ID      + YR  RHGK++P+ERD SNVP++  G  CT  + LN
Sbjct  415  DFMGCVLAWNTRNIDATSTGNYNYKYRYLRHGKLRPFERDISNVPFSPDGKPCTPPA-LN  473

Query  475  CYQPLKSYDFTDTVGIGYQPYRVVVLSFELLNAPATVCGPKQSTELVKNKCVNFNFSGLT  534
            CY PL  Y F  T GIGYQPYRVVVLSFELLNAPATVCGPK ST+L+KN+CVNFNF+GLT
```

```
Sbjct  474   CYWPLNDYGFYTTTGIGYQPYRVVVLSFELLNAPATVCGPKLSTDLIKNQCVNFNFNGLT  533

Query  535   GTGVLTDSTKRFQPFQQFGRDISDFTDSVRDPKTLQILDISPCSYGGVSVITPGTNASNV  594
             GTGVLT S+KRFQPFQQFGRD+SDFTDSVRDPKT +ILDISPCS+GGVSVITPGTNAS+
Sbjct  534   GTGVLTPSSKRFQPFQQFGRDVSDFTDSVRDPKTSEILDISPCSFGGVSVITPGTNASSE  593

Query  595   VAVLYQDVNCTDVPTMLHAEQVAHDWRVYAVNTDGNMFQTQAGCLVGATYDNTSYECDIP  654
             VAVLYQDVNCTDV T +HA+Q+   WR+Y+  T  N+FQTQAGCL+GA + +TSYECDIP
Sbjct  594   VAVLYQDVNCTDVSTAIHADQLTPAWRIYS--TGNNVFQTQAGCLIGAEHVDTSYECDIP  651

Query  655   IGAGVCAKFGSTKM----RQQSILAYTMSIGEDQSVAYSNNSIAIPTNFSISVTTEVLPV  710
             IGAG+CA + +  +    Q+SI+AYTMS+G D S+AYSNN+IAIPTNFSIS+TTEV+PV
Sbjct  652   IGAGICASYHTVSLLASTSQKSIVAYTMSLGADSSIAYSNNTIAIPTNFSISITTEVMPV  711

Query  711   SMTKTSVDCNMYICGDSTECSNLLLQYGSFCTQLNRALSGIAVEQDRNTRDVFAQTKTIY  770
             SM KTSVDCNMYICGDSTEC+NLLLQYGSFCTQLNRALSGIA EQDRNTR+VFAQ K +Y
Sbjct  712   SMAKTSVDCNMYICGDSTECANLLLQYGSFCTQLNRALSGIAAEQDRNTREVFAQVKQMY  771

Query  771   KTPNIKDFGGFNFSQILPDPGKPSQRSFIEDLLYNKVTLADPGFMKQYGDCLGGINARDL  830
             KTP +K FGGFNFSQILPDP KP++RSFIEDLL+NKVTLAD GFMKQYG+CLG INARDL
Sbjct  772   KTPTLKYFGGFNFSQILPDPLKPTKRSFIEDLLFNKVTLADAGFMKQYGECLGDINARDL  831

Query  831   ICAQKFNGLTVLPPLLTDDMIAAYTAALISgtatagytfgagaaLQIPFAMQMAYRFNGI  890
             ICAQKFNGLTVLPPLLTDDMIAAYTAAL+SGTATAG+TFGAGAALQIPFAMQMAYRFNGI
Sbjct  832   ICAQKFNGLTVLPPLLTDDMIAAYTAALVSGTATAGWTFGAGAALQIPFAMQMAYRFNGI  891

Query  891   GVTQNVLYENQKQIANQFNKAISQIQDSLTTTTTALGKLQDVINQNAQALNTLVKQLSSN  950
             GVTQNVLYENQKQIANQFNKAISQIQ+SLTTT+TALGKLQDV+NQNAQALNTLVKQLSSN
Sbjct  892   GVTQNVLYENQKQIANQFNKAISQIQESLTTTSTALGKLQDVVNQNAQALNTLVKQLSSN  951

Query  951   FGAISSVLNDILSRLDKVEAEVQIDRLITGRLQSLQTYVTQQLIRAAEIRASANLAATKM  1010
             FGAISSVLNDILSRLDKVEAEVQIDRLITGRLQSLQTYVTQQLIRAAEIRASANLAATKM
Sbjct  952   FGAISSVLNDILSRLDKVEAEVQIDRLITGRLQSLQTYVTQQLIRAAEIRASANLAATKM  1011

Query  1011  SECVLGQSKRVDFCGKGYHLMSFPQAAPHGVVFLHVTYVPSEQKNFTTAPAICHNGKAYF  1070
             SECVLGQSKRVDFCGKGYHLMSFPQAAPHGVVFLHVTYVPS+++NFTTAPAICH GKAYF
Sbjct  1012  SECVLGQSKRVDFCGKGYHLMSFPQAAPHGVVFLHVTYVPSQERNFTTAPAICHEGKAYF  1071

Query  1071  PREGVFVMNGTHWFITQRNFYSPQVITTDNTFESGTCDVVIGIVNNTVYDPLQPELESFK  1130
             PREGVFV NGT WFITQRNF+SPQ+ITTDNTF SG CDVVIGI+NNTVYDPLQPEL+SFK
Sbjct  1072  PREGVFVFNGTSWFITQRNFFSPQIITTDNTFVSGNCDVVIGIINNTVYDPLQPELDSFK  1131

Query  1131  QELDKYFKNHTSPDVDLGDISGINASVVDIKKEIEHLNEIAKNLNESLIDLQELGKYEQY  1190
             +ELDKYFKNHTSPDVDLGDISGINASVV+I+KEI+ LNE+AKNLNESLIDLQELGKYEQY
Sbjct  1132  EELDKYFKNHTSPDVDLGDISGINASVVNIQKEIDRLNEVAKNLNESLIDLQELGKYEQY  1191

Query  1191  IKWPW  1195
             IKWPW
Sbjct  1192  IKWPW  1196
```

Figure S4. Sequence alignment of the wild and the novel spike proteins.

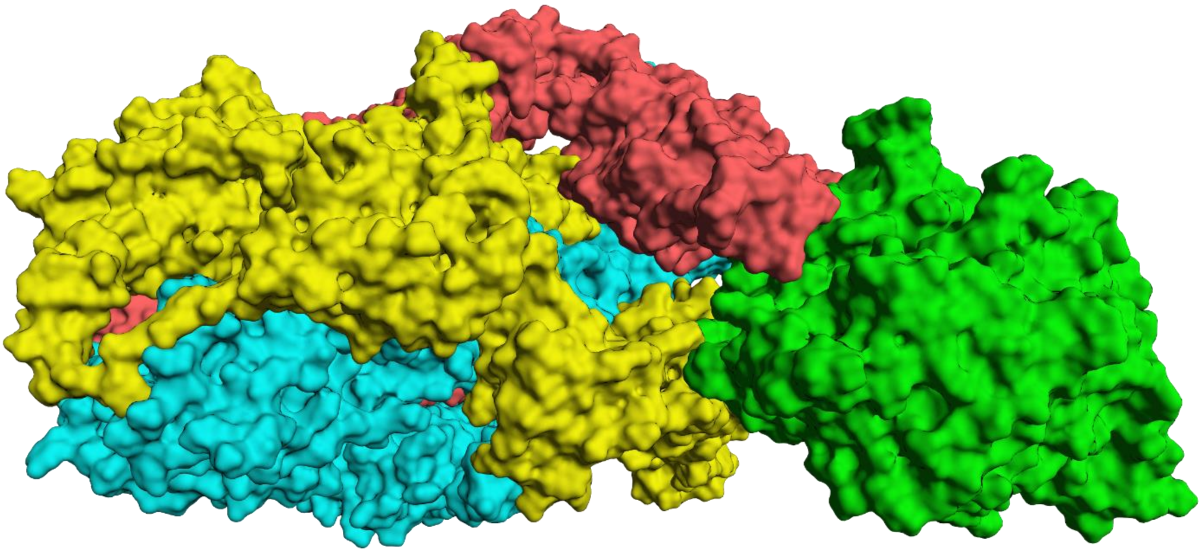

Figure S5. wild-ACE (green) interaction.

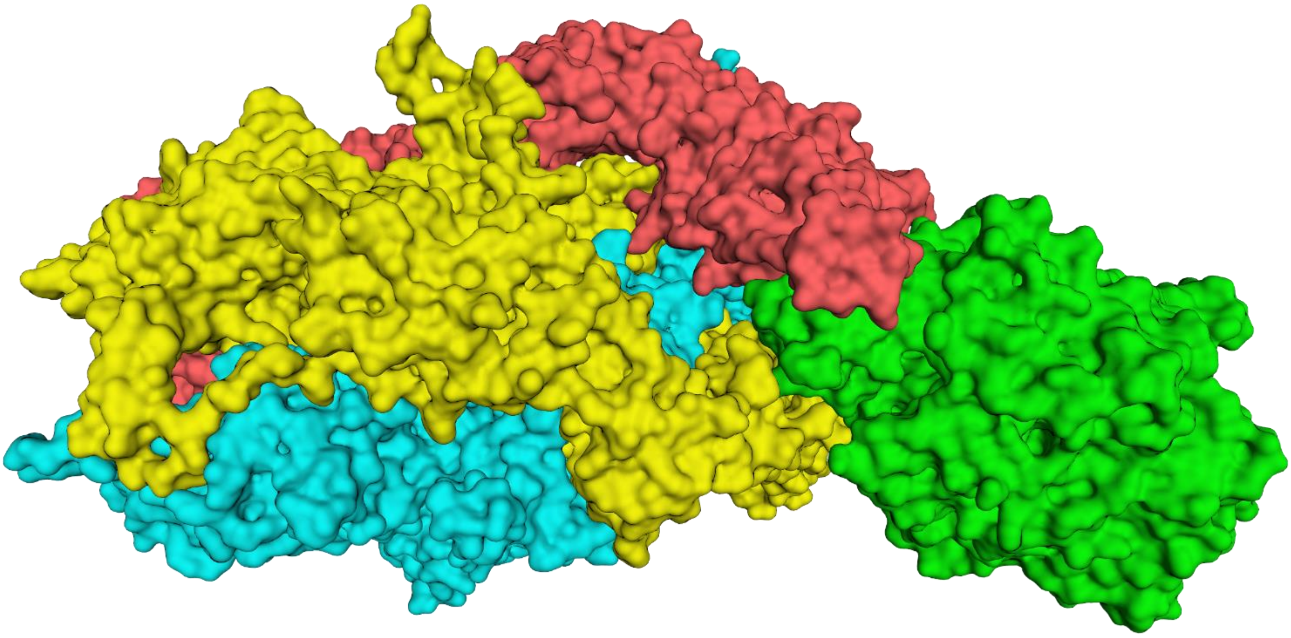

Figure S6. Novel-ACE2 (green) interaction.

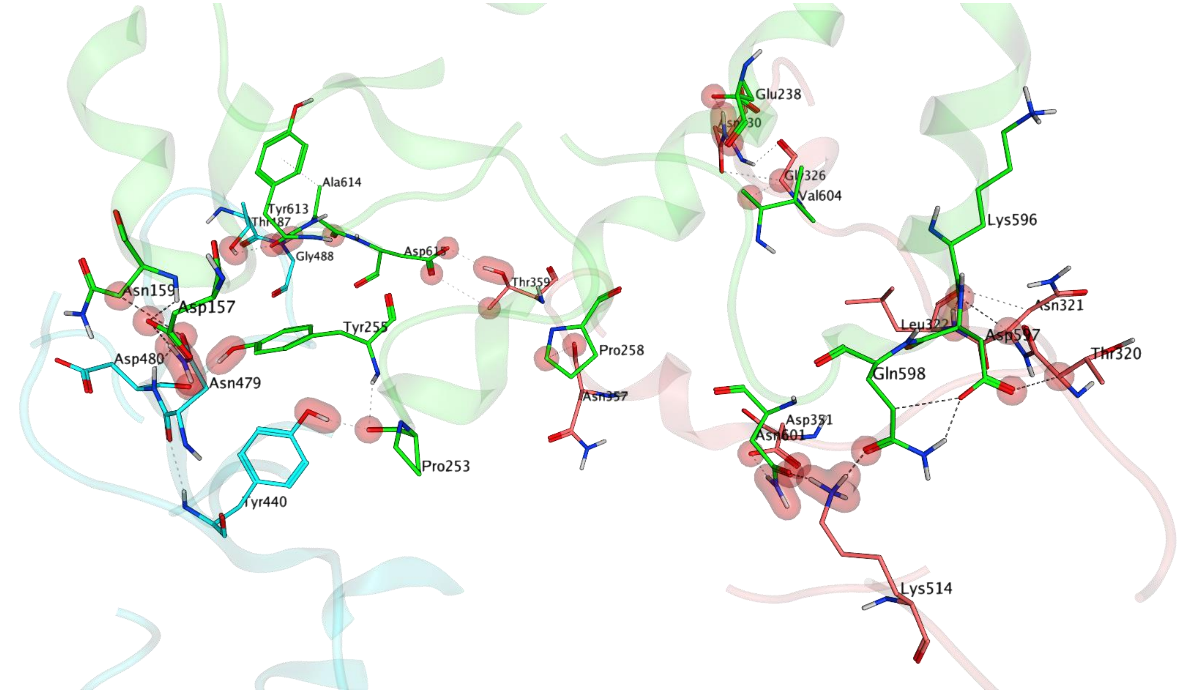

Figure S7. Wild-ACE2 interactions.

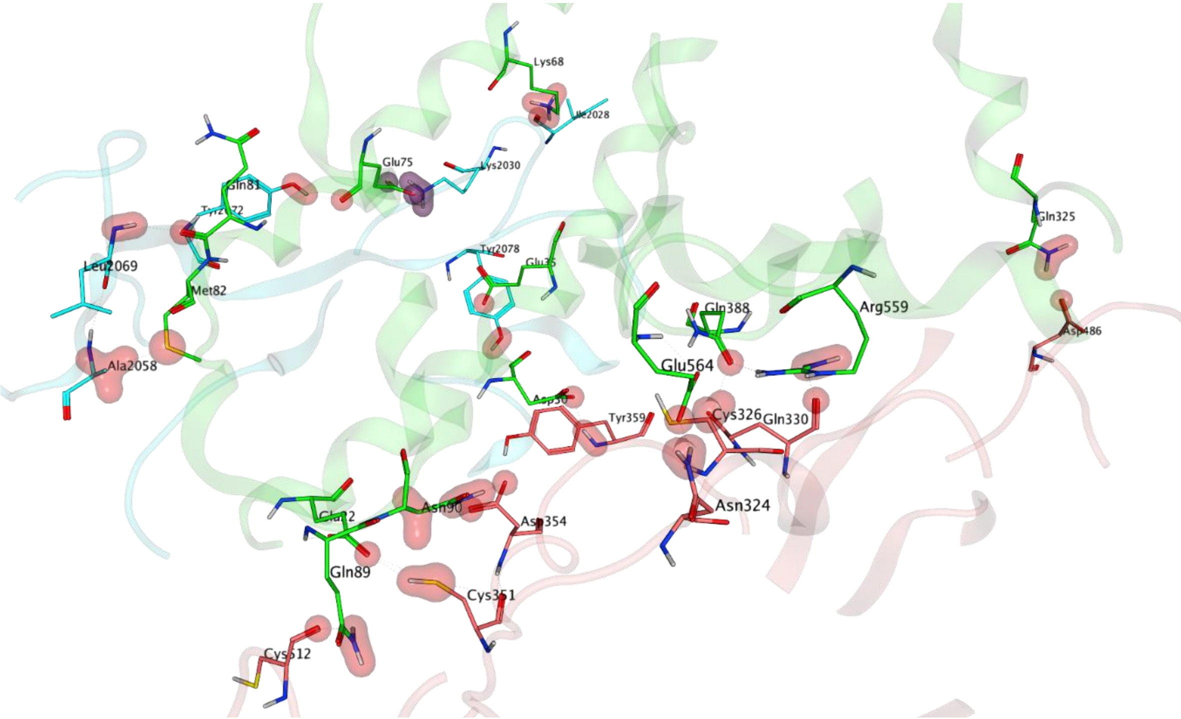

Figure S8. Novel-ACE2 interactions.
